# The potential of educational comics as a health information medium

**DOI:** 10.1111/hir.12145

**Published:** 2016-06-13

**Authors:** Sarah McNicol

**Affiliations:** ^1^ Education and Social Research InstituteManchester Metropolitan UniversityCreweUK

**Keywords:** bibliotherapy, consumer health information, health information needs, information sources, interviews, learning, patient information, qualitative research

## Abstract

**Objectives:**

To investigate ways in which educational comics might provide support in dealing with feelings and attitudes towards health conditions, as well as improving understanding of factual information and to identify potential weakness of comics as a medium for health information.

**Methods:**

Semi‐structured interviewees with eleven university students who either had a mental or physical health condition themselves or had a family member with a health condition.

**Results:**

The result highlighted the potential value of comics as a format for health information. In addition to conveying factual information, comics offer opportunities for self‐awareness, reassurance, empathy, companionship and a means to explore the impact of illness on family relationships. However, there are notable barriers to the greater use of comics to provide health information, namely, a lack of awareness of, and easy access to, educational comics, along with the perception that comics are exclusively light‐hearted and for children.

**Conclusions:**

Currently, the full potential of comics in health settings is not being realised. Health information professionals may be in a position to address this issue through identifying, cataloguing, indexing and promoting comics as a legitimate format for health information.

**Key Messages**

- Narrative, characterisation and images are key features of comics that may be important when using this medium for health information purposes.
- Comics can offer patients and family members opportunities for self‐awareness, reassurance, empathy, companionship and ways to explore the impact of illness on family relationships.
- The potential impact of visual and textual metaphors in health comics is complex and needs to be explored through further research.
- Health information workers are well positioned to help improve awareness of, and access to, health comics and to highlight their potential role in health care.
- Through drawing attention to their use as information sources, information workers can help to change perceptions of comics as being merely frivolous and for children.

## Background

Educational comics can be defined as, ‘a subset of comics whose purpose is not to tell a story or entertain but to transfer information or communicate concepts’.^1^ In the field of health information, educational comics may have a number of purposes, including raising awareness (for example, of disease symptoms); preparing patients (for instance what to expect from a medical procedure); assisting with decision‐making (such as deciding between treatment options); promoting self‐management of chronic conditions; or simply increasing understanding and acceptance of a condition. As described on the Graphic Medicine website, there can be a variety of reasons for the choice of comics as a health information medium from a desire to push the boundaries of what comics can do to a means to engage patients with low literacy levels. While the role of bibliotherapy has a long history^2^, ^3^, ^4^ and has been subject to considerable interest in recent years,^5^, ^6^, ^7^ less attention has been paid to the specific role of comics as therapeutic reading.

As McAllister points out, comics have the potential not merely to relay facts, such as risk factors for a condition, but also to deal with social issues surrounding illness.^8^ Educational comics may help patients, and their families, to better understand the fears, anxieties and expectations they can face by encouraging empathy as the reader relates to the events and characters in the story and connects these to their experiences. Therefore, while it is important to evaluate the factual comprehension of educational health comics, it is also essential to consider how reading a comic may impact on a patient's mental state, for example, whether they feel more positive about their condition and life in general; whether the story has led them to rethink their relationships with friends and family or health care professionals; whether any of their fears have been allayed; or whether their attitude towards self‐management or prevention has altered. To date, however, research into the effectiveness of comics as health information resources predominately consists of studies administering pre‐ and post‐intervention questionnaires to determine information learnt, and whether this information is retained and results in changes in behaviour over a period of time.^9^, ^10^, ^11^ This study attempts to start to redress the balance by focusing on the potential impacts of reading educational health comics on emotions and social relationships.

## Reading comics

Reading a comic requires the interpretation of not only text, but also images as the reader must negotiate two systems of codes which sometimes function independently, and at other times interact. As a hybrid word‐image format, therefore, comics have ‘dual narrative tracks’,^12^ which require the reader to develop a number of strategies to make sense of the various possibilities presented. Thus, comics do not present a single, indisputable message, but instead rely on the reader to produce their own interpretation as they create an overall meaning by relating both the words and images of the comic to their own experiences. Each reader individualises their response, meaning that comics are often characterised by the presence of multiple messages involving the co‐presence and interaction of visual and linguistic codes. As a result, there is no single ‘correct’ or absolute meaning, but rather more or less equally valid alternative interpretations. This process is described in greater detail elsewhere.^13^ At first sight, this ‘plurality of messages’^14^ might seem to be a disadvantage for a resource intended to convey health information, but there is, of course, a great deal of uncertainty surrounding illness; two patients with the same illness can display different symptoms and respond differently to treatments. Furthermore, each patient responds to an illness as an individual, with different reactions to diagnosis and differing priorities when undergoing treatment for example. It is precisely the ambiguity of comics which can help to capture this diversity of experiences.

## Objectives and methods

The principal aims of this small‐scale research project were to investigate the ways in which educational comics might provide support in dealing with feelings and attitudes towards health conditions, as well as improving understanding of factual information, and to identify any potential weaknesses comics may have as a medium of health information.

Potential interviewees were identified with the help of tutors and course administrators who circulated information about the project to their students from across Heath, Education and Information Studies faculties or departments at [Manchester Metropolitan University UK]. Those who were interested in taking part were asked to complete a short online screening proforma. From volunteers listing a condition for which two or more educational comics could be sourced, eleven participants were purposively selected to ensure a mix of gender and age as far as possible and to include those with a condition themselves as well as those who had a family member with a health condition. In total, one man and ten women were interviewed. As would be expected among university students, interviewees were young adults. Seven were aged between 18 and 24 and four were between 25 and 34 years. Five interviewees had a health condition themselves and six had a relative (parent or grandparent) with a condition. The interviews focused on comics for eight different health conditions, including both mental and physical illnesses (namely, anxiety, depression, testicular cancer, Crohn's disease, diabetes, dementia, lupus and MS). Further information about the participants and comics used is provided in the supplementary materials.

A semi‐structured interview, lasting approximately 45 minutes, was conducted with each participant. Interviewees were asked to read between two and four comics relating to their condition prior to the interview. While educational comics are produced, primarily, for educational purposes, medical narratives or illness accounts are graphic novels and comics produced with more literary or artistic intentions. However, the boundaries between the two are inevitably blurred and some of the titles selected for this study may be considered to be medical narratives by some readers. The criterion for inclusion in the study was that the comics should contain an educational or informational aim that could be argued to be equal to, or greater than, their artistic or literary purpose. These comics were then used as stimulus material during the interview which included questions about the interviewees’ views of comics and access to health information in general, as well as their responses to the comics provided and to the format more generally as a source of health information. Interviews were audio‐recorded, transcribed (intelligent verbatim), anonymised and analysed thematically using Nvivo. An inductive approach was taken with initial codes devised from a first reading of the transcripts and codes added or combined to take account of additional themes which emerged from a closer reading.

## Results

**Figure 1:**
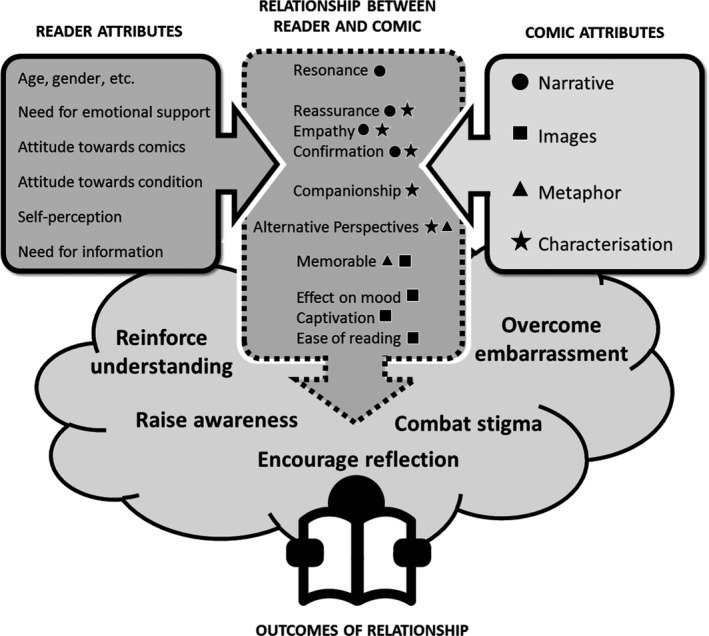
Relationship between the comic and the reader (design courtesy of Peter Whitton, Manchester Metropolitan University)

Two of the main themes to emerge from the interviews were the effects of narrative and characterisation and the particular role of images in comics. These topics frequently led onto discussions about the possible social and emotional benefits of educational health comics. See Fig. 1 for a visual representation of the links between these effects and beneficial outcomes. However, there were also potential problems identified which may act as a barrier to the wider use of comics as a form of health information.

## The effects of narrative and characterisation

Interviewees agreed that presenting health information within a narrative could make a complex topic easier to understand and may mean that people would be able to relate to what was happening more readily:

It's much more relatable when there's scenarios and everyday situations compared to the Internet where you can't imagine…cells are dying or whatever. You can't picture that, but you can picture yourself in a situation.

(Interviewee D)

Interviewees responded positively to most of the characters in the stories; they were able to empathise with their situations and some suggested that this personal link would help them to relate to, respond to and remember the information better than might have been the case if it was presented in a more abstract way:

I can relate to being a child and the child saying those things and the mum's response and thinking, ‘That's my mum would have said; that's what she said to us when we were younger’.

(Interviewee C)

… I think it is the best way to do it ‘cos if you link yourself with someone, you remember it better then. If you link yourself with the character you remember about the information that's being given to you.

(Interviewee A)

However, factors such as age and gender sometimes affected the extent to which interviewees were able to fully relate to the characters and narratives presented in the comics provided:

These guys seem to be quite young and just starting out and they're worried about going to the pub and things like that. I don't have those concerns; I have different concerns about going to work…

(Interviewee B)

As well as helping interviewees to better understand their own situation, reading the comics could also mean they were able to empathise with others with the same condition, but in different circumstances. Reading the comics, therefore, provided patients with an opportunity to view their condition from a different perspective:

Describing loneliness…a lonely fog…being mixed up. I never really saw it like that…It's never a fog, that I'm trying to get out of, it's more that I'm trying to keep up…I couldn't really identify with that…but even so, it was good to see the difference, to understand what it was like for someone else…

(Interviewee G)

The potential for empathy through relating to the characters and their experiences was also a common theme in interviews with people who had a relative with a particular condition:

It did make me think more about my dad's experience at that age, ‘cos he was at university when he was diagnosed, so the impact that would have had on him at that point, how that would have changed…

(Interviewee E)

Related to this idea of empathy was the notion that comics could provide a sense of companionship, reassurance and recognition through the realisation that others were dealing with the same issues:

I could really emphasise with her. I've gone through some of those things and I thought, ‘Gosh, yeah’.. I didn't realise I had it at the time, what she's talking about… So with me, it was…a nice confirming thing that there are other people…I've had CBT as well so I smiled to myself when I got to that bit because I thought, ‘Yeah, that's the kind of thing that I stand there and do as well’, it was nice.

(Interviewee J)

## The role of images

The role of images within the comics was clearly important to interviewees. Comments indicated that images often appeared to be more powerful in conveying the author's message than the words used:

I think you're drawn to the pictures more than what's being said…

(Interviewee F)

Interviewees described how the use of images could offer comics a number of advantages over written health information materials. Firstly, images could make the resources more interesting and potentially easier to read, especially for people who may be distressed or tired due to illness.

It just makes it a bit more interesting to read. And people will see that on the shelves and think, ‘Oh, there's images…’…whereas if you see all the writing, you think, ‘Phew, I don't know if I could go through all that writing now’.

(Interviewee A)

Images could also be used to support, or reinforce, written explanations by presenting the information in a different way, and one which might resonate more strongly with the reader:

…if I was on a website and I read ‘Patients with lewy bodies may experience hallucinations’, I might…’Okay then’ and move on, but this made me imagine it quite a bit more, so it gave me quite a bit more insight into it.

(Interviewee D)

In particular, interviews thought that the types of visual metaphors and analogies presented in comics could be helpful in explaining complex ideas in an easily understandable and memorable way. For example, commenting on a title from the Medikidz series which represents the body as a planet inhabited by various characters, one interviewee said:

I think that's definitely easier; if it was one like that which was trying to explain what it was using these different characters for different parts of the body, it's good actually to get your head around it…

(Interviewee C)

While some metaphors promoted immediate recognition and a sense of empathy, others presented interviewees with alternative ways of viewing an aspect of their condition, prompting them to reflect on, and re‐examine, their own understandings:

I never personally thought of that, but I can imagine it is…you're carrying a whole load of weights in a back pack and things like that. So visually it's useful. Because I think it's quite powerful when you see this blob hanging on to this person…straightaway you know there's something weighing that person down…

(Interviewee J)

However, images could also have a negative impact, especially as, in some case, the images were closely linked to mood and feeling by interviewees. For example, some described how dark or confusing images could have a negative impact on their mood:

Both these two feel quite dark in terms of psyche as well as being in black and white. These feel a bit more down hearted, it's all a bit doom, gloom and despair …

(Interviewee B)

## Potential social and emotional benefits of educational comics

Interviewees in this study identified a number of social and emotional benefits that could arise from the use of narrative, characterisation and images in health comics. Comics could offer reassurance; lead to increased self‐awareness; help to raise awareness of a condition; or be used to open up a discussion with health care professionals or family members. For most interviewees, there was some element of reassurance, or confirmation, to be gained from reading the comics:

I suppose it is just being positive in terms of confirming rather than anything else.

(Interviewee J)

A lot of the time…I was nodding my head a lot, ‘Yeah, yeah, yeah…’

(Interviewee G)

However, reading the comics was not always an easy process for interviewees; the narratives could raise sensitive issues and lead to distress and concern. On occasions, this could be therapeutic, helping interviewees to develop a better understanding of their feelings, for example, but it is also possible that, for some, comics could provoke concerns without offering any resolution:

That was very hard for me when I was reading those comics to realise that I cannot get rid of it…When I was reading they caused me a lot of bad feelings…stress, but the benefits were more…

(Interviewee K)

While interviewees were keen that comics should avoid trite clichés, they felt it was important for the overall message presented to be a positive one to demonstrate that that living with a health condition could have positive, as well as negative, aspects:

If people read that and they start thinking, ‘Oooh, uh‐oh…’ It's all bad news, but there's obviously good things as well that can happen…Maybe a bit more about what the good things were rather than just the bad.

(Interviewee A)

As most interviewees felt they already had a good understanding of their condition, it is perhaps unsurprising that they did not feel they had gained significant additional factual knowledge through reading the comics. However, comments made by many suggest they did gain increased self‐awareness of their own actions and responses to health issues, and of the ways in which they were currently managing their condition:

It just brought it back to my awareness because I live with it all the time I don't even think about it. It helped in that way…it brought things up into my mind that were in my mind but…suppressed… It made me realise that I'm doing alright considering…I've got all this going on as well, I just don't realise that I'm doing it. It made me think, ‘Yeah I've got it and this is what I'm facing’…

(Interviewee B)

Interviewees commented on the potential role of educational comics in raising awareness of issues around particular conditions, especially those which were less well known or may be subject to stigma:

…possibly things where there's more stigma…like mental health conditions and things like that, maybe there's more of a role for comics…Something where you could start with the preconceptions and then tease them out…

(Interviewee E)

Several interviewees commented that, not only were the comics relevant to people currently suffering from a condition, but they may be of interest among the wider population too:

It's a good way to get information across actually…Those are really, really good if they could be directed around the general public.

(Interviewee G)

A theme to emerge strongly was the potential of comics to help family and friends of the person with a health condition to better understand what they were experiencing and feeling and why they might behave in certain ways. Interviewees believed comics might be helpful for this purpose because they are accessible, easy and enjoyable for someone to read and because the combination of words and images might put across a point more effectively than text alone. They might also be helpful to approach a topic which was difficult or embarrassing to discuss:

I'd quite like to use them to help share with other people what I'm going through more than them helping me and I would think they're be useful as a tool to tell other people, ‘cos I find it sometimes…I can tell someone I'm tired ‘til I'm blue in the face, but they, ‘Well stop being tired then!’. They don't understand that it's part of my condition…

(Interviewee B)

…my mum doesn't like to tell people how she's feeling, so a lot of people in the family just wouldn't know. So I think this is quite a good thing, say, ‘I've got this, just have a read of it’ and you make a mental note, ‘Oh, that's why they're like that on certain days’… this gives you an insight into behind the scenes where she might keep that back from family members…

(Interviewee C)

Interviewees felt that comics might have a role at various stages in the ‘patient journey’. Of course, this depended to some extent on the issues represented in the titles they had read. Overall, there was support for the use of some of the comics around the time of diagnosis, although opinions differed as to whether comics might be useful immediately upon diagnosis or a little while after. The critical consideration, as several interviewees pointed out, was the needs and preferences of the patient themselves.

## Potential problems of educational comics

While many positive aspects of comics were discussed during the interviews, there were also a number of issues that might prove problematic in the more widespread use of comics as a health information medium. The two main concerns identified from this research were poor access to comics about health conditions and negative perceptions based on the limited previous experience of comics dealing with serious issues.

## Access and availability of educational health comics

Educational comics can be found about a wide range of illnesses. However, some conditions have undoubtedly received more attention from comic book creators than others. In attempting to source materials for this study, it became apparent that although a number of comics were available for some conditions, for example diabetes, anxiety and depression, for others, it was not possible to locate any suitable materials.

Although most interviewees had read comics as children and a few were still occasional comics readers, none had a particularly strong interest in the medium. While they had been active in seeking out health information, none of the interviewees were aware of educational comics before taking part in the project and most had never read a comic about a ‘serious’ issue before:

It's interesting, I've never seen books like that, or anything to do health, never noticed anything presented in a comic form ever, so it's the first time that I had encountered that… I had never thought that they would be used in this way.

(Interviewee J)

## Initial perceptions

For many interviewees, their understanding of what a comic typically looks like was based on superhero and children's comics. The materials they had been presented with for this project did not, therefore, always match their expectations of the format and this sometimes caused a little confusion or uncertainty:

…the word comic in itself…You say the word comic…pretty much all of them will think Marvel or DC or something like that…or light‐hearted comedy…This is a comic about depression, then people would think, ‘That's an oxymoron surely. What's going on?’

(Interviewee G)

Based on the previous experience of comics as being light‐hearted and trivial, some interviewees were uncertain as to whether the medium would be able to deal with serious health‐related issues effectively:

I wonder if such a serious issue can seriously be covered within a cartoon. I'm kind of a bit mixed on that, whether it works well or whether it doesn't. Does it belittle the issue because it's within a cartoon format, or is it appropriate? I honestly don't know what I feel about that…

(Interviewee J)

Furthermore, rather than helping to put across information more effectively, some interviewees felt that the images might detract from the serious message the comic was trying to convey:

…you lose the message of what they're talking about because there's so much going on in the pictures I think…they'll just be distracted, there's so much going on…would you be able to get through to them that they're talking about quite serious things inside someone's body? I'm not sure.

(Interviewee B)

However, after being asked to read several comics to take part in this research, there was evidence that a number of interviewees had begun to change their attitudes towards comics; in particular, they showed more recognition of their potential as an accessible, appealing source of health information:

…that comics can be more informative because in the past I've just seen them for entertainment, but I definitely think they can be more informative and be used in a really positive way…portraying serious information, but in a light‐hearted sense.

(Interviewee D)

Furthermore, a few interviewees commented that regular comics readers they knew may more readily embrace the use of this medium for health information:

…my husband, he's into computer games and stuff; he probably would be quite interested in these rather than…some stuff I printed off Google…He's more likely to read it then maybe ask me questions about it.

(Interviewee B)

## Discussion

The interviews conducted for this project have highlighted the potential value of comics as a format for health information. Comics potentially offer opportunities for self‐awareness, reassurance, empathy, companionship and a safe and neutral way to explore the impact of illness on family relationships. While a straightforward patient information leaflet can convey factual information, a comic can also help patients, and relatives, to understand much more about the fears, anxieties and expectations a patient may need to deal with. Crucially comics are able to convey this information not as generic statements, but by encouraging empathy as the reader relates to the characters and narrative. Readers may, thus, gain greater insight into their own feelings and reactions. These findings support the view of comics as ‘a very non‐threatening medium’ as well as a ‘personalising medium’,^8^ and one which ‘universalises the illness experience’^15^ meaning they ‘may be a very effective tool in creating empathy and compassion’^8^. Interviewees reported that key features of comics, such as the use of images, characterisation and narrative could not only make complex issues easier to understand, but they also enable the reader to relate more closely to the conditions being discussed, allowing them to either gain greater self‐awareness of their own situation or to better empathise with family members. However, it is important to note that the findings from this research refer only to short‐term reactions to reading comics and further research is needed to investigate the possible longer term effects of comics on the social and emotional aspects of illness.

Green and Myers have previously hypothesised that comics may make patients feel ‘more focused and in control’ and ‘less isolated and more hopeful’.^15^ To an extent, these ideas have been borne out in this research. Many interviewees felt reading the comics gave them greater self‐awareness and understanding of their condition and its impact on their lives. The notion of companionship, which can be viewed as a combination of empathy, recognition and reassurance, was another strong theme and interviewees frequently commented that reading the comics helped to make them aware they were not alone and that there were other people dealing with the same issues. However, the extent to which the comics selected made interviewees more hopeful varied; reading about distressing aspects of their condition could cause concern and several interviewees felt that some of the titles they had been given, and especially the images used, presented an overly pessimistic view. The majority of those who discussed this issue said they would like comics to present the more positive aspects of illness, as well as the problems, and felt it was important that this outlook was reflected in the visual representations used as these could have a strong and immediate impact on the reader.

While research has shown the use of pictures or cartoons in patient information to be more effective than using text alone, these studies have usually focused on effects such as recall, attention and comprehension of factual information.^16^ The use of images and visual metaphors in health comics, in particular, their social and emotional impacts, is a topic in need of greater exploration. Metaphors allow us ‘to understand abstract areas of our lives in terms of more concrete and embodied experiences’.^17^ Many of the metaphors in the comics used in this study represented conventional cultural metaphors in a graphic form, for example illness as a fight or as a burden, which interviewees could recognise and empathise with. However, other metaphors did not simply reinforce conventional representations of illness, rather, they prompted interviewees to question their views of their own (or their relative's) condition and helped them to consider alternative viewpoints. In this way, the comics can be argued to deepen and test a reader's understanding of their condition, not merely reinforce their pre‐existing conceptions. However, some images prompted mixed feelings from interviewees: while they believed that images, especially visual metaphors, could be powerful in explaining more issues more clearly and in a more immediate way, the presence of images in serious information sources intended for adults could be unsettling.

As Williams describes, graphic medical narratives are usually consumed by readers with ‘some sort of vested interest’.^18^ However, some interviewees in this study felt that the comics they had been given to read could have a wider appeal. Perhaps, as Spiegel *et al*. and Brown *et al*. have argued in the case of science and public health comics, respectively, comics can enable non‐specialists to engage with complex topics.^19^, ^20^

This research has also highlighted potential issues around the use of health information comics. A lack of awareness of these types of resources was identified as a potential barrier to their wider use; interviewees were unsure where they might find educational comics about their condition. In the early 1990s, Rifas referred to the ‘perennially disorganised state of educational cartooning’^21^ and this is still true today. While it is a relatively simple matter to locate educational comics with ISBN numbers, many more ephemeral publications are not listed in standard bookseller catalogues and remain unknown even to those who have actively sought information about a condition for many years. Health information workers clearly have a role here in helping to make health information comics more visible for their users, and in investigating ways to integrate comics within databases and other resources commonly used to identify more traditional health information materials.

A further problem was the general perception of comics as being light‐hearted and for children. There is a danger that the effectiveness of comics as a health information medium may be limited if readers are less likely to take information provided via this format seriously. Such ingrained views of comics could prove a challenge to the wider use comics as a credible source of health information, especially for adults. As the format has most commonly been seen as a technique to make information accessible for low literacy patients^22^, ^23^ or for young people or non‐native speakers^15^, it is unsurprising that the full potential of comics as a source of health information for the general population has not been widely recognised. However, this project has shown that people can quickly modify their views of comics and adopt much more positive attitudes after reading examples of relevant educational comics. Health information workers may also have a role to play here in selecting appropriate comics to promote to their users and thereby encouraging the acceptance of comics as credible information sources.

## Conclusions

There are, of course, limits to this small scoping study. None of the volunteers were regular comics readers; all were fairly highly educated; and the majority were younger and female. Nevertheless, this research has indicated that there are potential benefits to using comics not simply to communicate factual health information about a wide range of physical and mental conditions, but to provide patients and family members with greater understanding of the social and psychological aspects of illness through the use of narrative, characterisation and images. However, a lack of awareness of their existence and knowledge of how to access health‐related comics, as well as pre‐existing attitudes towards comics as frivolous and aimed at children, are issues to be overcome if educational comics are to become more widely accepted. Health information professionals have a key role to play in identifying, cataloguing, indexing and promoting educational health comics. This could not only help to improve knowledge of, and access to, health information in a comics format, but it could also contribute to changing perceptions and encouraging both patients and family members, as well as health care workers, to view comics as a credible and potentially valuable form of health information.

## Appendix

**Figure A1** List of comics used:

1. Abram, D. *Worry Wart*. Razarhawk, 2014
2. Ankerman, D. & Seliutina, Y. *My MS Story Reduced to a Comic,* 2014. Accessible at: http://www.healthline.com/health/multiple-sclerosis/diagnosis-story (accessed 4 Feb 2015).
3. Brosh, A. *Hyperbole and a Half: Depression Part 2,* 2013. Accessible at: http://hyperboleandahalf.blogspot.co.uk/2013/05/depression-part-two.html (accessed 4 Feb 2015).
4. C.F. *Face It,* 2013. Accessible at: http://opinionator.blogs.nytimes.com/2013/02/18/face-it/?_r=0 (accessed 4 Feb 2015).
5. Chilman‐Blair K. & DeLoache, S. *Medikidz Explain Crohn's Disease: What's up with Keaton?* London: Medikidz Ltd., 2014
6. Chilman‐Blair K. & DeLoache, S. *Medikidz Explain Type 1 Diabetes: What's up with Ashleigh?* London: Medikidz Ltd., 2012.
7. Chilman‐Blair K. & Rimmer, I. *Medikidz Explain Multiple Sclerosis: What's up with Ryan's Mum?* London: Medikidz Ltd., 2014.
8. Clay. *Depression Comix,* 2012‐14. Accessible at: http://www.depressioncomix.com/posts/characters/depressed-character-04/ (accessed 4 Feb 2015).
9. Demetris, A. *Dad's Not All There Anymore,* 2012.
10. Dwyer, J. & Torres, T. (n.d.). *Insu‐man: The origins of the diabetes superhero*. Accessible at: http://static.squarespace.com/static/53e3bacbe4b022bcdbe1f538/t/542570dfe4b00bb1076d8f8b/1411739871303/Insuman%20Comic.pdf (accessed 4 Feb 2015).
11. Humberstone, T. *Everything You Never Wanted to Know about Crohn's Disease*, 2009. Accessible at: http://ventedspleen.com/blog/2007/10/22/24-hour-comic-everything-you-never-wanted-to-know-about-crohns-disease/ (accessed 4 Feb 2015).
12. Husband, T. *Take Care, Son*, 2014. Accessible at: http://www.dailymail.co.uk/health/article-2623800/The-saddest-goodbye-Endless-words-written-dementia-But-ANY-match-poignancy-cartoonist-Tony-Husbands-account-watching-steal-away-father.html(accessed 5 Feb 2015).
13. Keeper, M. *I Have Crohn's Disease*, 2012. Accessible at: http://www.graphicmedicine.org/mike-keepers-i-have-crohns-disease/ (accessed 4 Feb 2015).
14. King, M. & Martinot, C. *Someone You Know has MS: A book for families*, 2012. Accessible at: http://www.nationalmssociety.org/NationalMSSociety/media/MSNationalFiles/Brochures/Brochure-Someone-You-Know-has-MS_-A-Book-for-Families.pdf (accessed 4 Feb 2015).
15. Kolin, D., Brame, D., Chung, P. & Nyhof‐Young, J. *Screening and Diagnosis: Get on the ball*! University Health Network, 2005.
16. Leamy, C. *Diabetes is after your Dick!* 2012. Accessible at: http://www.metrokitty.com/comics/webcomics/diabdick/ (accessed 15 Jan 2015).
17. Learning About Diabetes. *My New Shadow*, 2012. Accessible at: http://www.learningaboutdiabetes.org/wp-content/uploads/pdfs-type_1_diabetes/MyShadowsPart5Full_COMPLETE.pdf (accessed 4 Feb 2015).
18. Learning About Diabetes. *The Mysterious Symptoms*, 2007. Accessible at: http://www.learningaboutdiabetes.org/wp-content/uploads/pdfs-about_diabetes/MysteriousSignsEN.pdf (accessed 4 Feb 2015).
19. Liss, D., Sharpe, K., Wong, W. & Sotomayor, C. *Iron Man: Early Warnings #1*, 2014. Accessible at: http://reader.marvel.com/#/issue/29952/wl/1 (accessed 1 Mar 2015).
20. Maury, P. & Morgan, N. (n.d.). *‘Ask Me 3’: Ms Jenkins visits the lupus clinic*. Accessible at: http://thelupusinitiative.org/downloads/Askme3_V2_Full_10_06.pdf (accessed 4 Feb 2015).
21. Maury, P. & Morgan, N. (n.d.). *Living with Lupus: Jackie learns how to live with lupus*. Accessible at: http://thelupusinitiative.org/downloads/Living_with_Lupus.pdf (accessed 4 Feb 2015).
22. Moen, E. *A Deep Comic about Depression, inspired by Robin Williams,* 2014. Accessible at: http://www.everfunny.com/a-deep-comic-about-depression-inspired-by-robin-williams/ (accessed 4 Feb 2015).
23. Nyhof‐Young J, Gooi P, Verbeeten K, Kim B. *A Courageous Journey: Experiences with Testicular Cancer*, 2005. Accessible at: http://dfcmopen.com/item/testicular-cancer-screen-diagnosis/ (accessed 22 Mar 2015).
24. Russell, M. *The Anxious and the Depressed* [Kindle edition], 2013.
25. Salberg, S. *A Comic about Anxiety*, 2013. Accessible at: http://heymonster.tumblr.com/post/19350033745/a-comic-about-anxiety (accessed 4 Feb 2015).
26. Sanchez Corral, A. (n.d.). *Mary and the Junk Food Eaters*. Accessible at: http://www.borderhealth.org/files/res_1904.pdf (accessed 4 Feb 2015).

**Table 1:** Information about participants

Interviewee	Age	Gender	Relationship to person with a health condition	Condition	Comics
A	18–21	Male	My mother/father	Testicular cancer	15, 23
B	25–34	Female	Myself	Crohn's Disease	5, 11, 13
C	18–21	Female	My mother/father	Lupus	20, 21
D	18–21	Female	Grandfather/grandmother	Dementia	9, 12
E	25–34	Female	My mother/father	Type 1 diabetes	6, 10, 17
F	18–21	Female	My mother/father	MS	2, 7, 14
G	18–21	Female	Myself	Depression	3, 8, 22
H	18–21	Female	Myself	Type 1 diabetes	6, 10, 17
I	22–24	Female	My father/grandparent	Type 2 diabetes	16, 18, 19, 26
J	25–34	Female	Myself	Anxiety	1, 4, 22, 25
K	25–34	Female	Myself	Anxiety	1, 4, 24, 25
